# Löwenstein-Buschke: Clinicopathologic Analysis of 78 Cases of Large and Giant Condyloma Acuminata of the Anus

**DOI:** 10.5146/tjpath.2020.01508

**Published:** 2021-01-15

**Authors:** Orhun Cig Taskın, Burcin Pehlivanoglu, Michelle D. Reid, Theodore Friedman, Michael Lee, Talaat S. Tadros, Sudeshna Bandyopadhyay, Josephine Akinfolarin, Ayse Armutlu, Olca Basturk, Volkan Adsay

**Affiliations:** Department of Pathology, Koç University Hospital, Istanbul, Turkey; Department of Pathology and Laboratory Medicine, Emory University School of Medicine, Atlanta, GA, USA; Department of Pathology, Wayne State University, Detroit, MI, USA; Department of Pathology, Memorial Sloan Kettering Cancer Center, New York, NY, USA

**Keywords:** Condyloma acuminata, Giant, Anus, Buschke-Lowenstein, Squamous cell carcinoma

## Abstract

***Objective:*** The nature and clinicopathologic associations of Löwenstein-Buschke disease are unclear.

***Materials and Methods: *** 78 anal condylomatous lesions (≥2 cm) were analyzed. Cases were classified based on size as “medium-large”(2-5 cm, n=59), “large” (5-10 cm, n=13) and “giant” (>10 cm, n=6).

***Results: *** Patients were predominantly males (male/female=70/8). The mean age was 38 years (range:20-66). Two distinct lining types were recognized: 1) Epidermal type, typically lacking overt koilocytotic change, with associated invasive carcinoma in 8%; 2) Mucosal type, often manifesting koilocytotic change, with associated invasive carcinoma in 21%. Three types of high-grade dysplasia were discerned: 1) Basaloid, 8/9 showing high-grade dysplasia/carcinoma in-situ but non-invasive lesions; 2) Keratinizing, innocuous-appearing, but 5/6 was associated with invasion; 3) Giant cell, showing scattered individual bizarre cells, with 3/5 showing invasive carcinoma. Overall, invasion was found in 14% of the cases. The bulbous, broad-based destructive pattern characterizing verrucous carcinomas of the upper aerodigestive tract was not observed. A statistically significant trend existed between the incidence of invasion and size: 8.5% for medium-large, 23% for large, and 50% for giant (p=0.02). There was no discernable trend in the depth of invasion relative to condyloma size.

***Conclusions: *** Our findings suggest that Löwenstein-Buschke lesions are mega versions of conventional condyloma. Being verrucoid, large and minimally invasive, they can be conceptually regarded as a form of verrucous carcinoma, but they do not display the histologic characteristics of verrucous carcinoma defined in the aerodigestive tract. They exhibit two types of linings: the mucosal type that often shows koilocytotic changes, and the epidermal type that can be difficult to recognize in biopsies. These lesions may be associated with invasive carcinoma, albeit limited in amount.

## INTRODUCTION

Anal condyloma acuminata refers to polypoid, cauliflower-shaped and pedunculated excrescences, histologically characterized by hyperkeratosis, surface parakeratosis and koilocytosis of the superficial cell layers (1,2). It is caused by infection with the human papillomavirus (2). It is frequently found in sexually active people, representing the most common sexually transmitted disease in the United States.

Giant condyloma acuminatum (GCA) of the anus, also called Giant Condyloma of Buschke and Löwenstein, was initially thought to be a larger version of the conventional condyloma with minimal biological aggressiveness. Later on, some authors emphasized that GCAs were distinguished from traditional condylomas (3,4). Although they were cytologically unremarkable, locally invasive features with transformation to squamous cell carcinoma were intermittently noted, characterizing GCA as a malignant tumor (5). Therefore, GCAs have been categorized as a distinct clinical entity, a “carcinoma-like condyloma,” with the propensity to locally invade and recur, but without the predilection to metastasize. It is classified as “verrucous carcinoma of anus” by some.

The definition of “giant”, on the other hand, has been highly variable, and controversy exists over the terminology, size cut-off, histology, and degree of malignant potential of this lesion. The incidence of conventional malignant change (carcinoma in-situ or invasive carcinoma) and the relationship of size with malignant potential in GCAs have not been thoroughly investigated. In this study, we analyzed the largest series of large condylomas of the anal region with the intent to determine the distinct histological features including patterns of dysplasia and correlate them with clinical and demographic data.

## MATERIAL and METHOD

### Case Selection and Classification Criteria

78 resected anal condylomas that measured 2 cm or more and were diagnosed between 1986 and 2007 in the authors’ institutional files were reviewed. Institutional review board approval was obtained. Data on patient demographics including age, gender, HIV status and clinical presentation were extracted from the medical records, including the pathology reports.

All of the cases were procedures performed with the goal of complete removal of the lesion. The cases were classified according to their size, arbitrarily, as medium-large (2-5 cm) (n=59, 75%), large (5-10 cm) (n=13, 17%) and giant (>10 cm) (n=6, 8%). The slides were reviewed to determine the histologic features of the condyloma, the cell types occurring in the lesion, including the presence of a granular layer and keratotic layer, and the type and the grade of dysplasia.

For the purposes of this study, the high-grade squamous intraepithelial lesions were regarded in two groups: 1) High-grade dysplasia and 2) frank carcinoma in-situ, where there is florid atypia and pattern that raises the concern for invasive carcinoma, but no definitive invasion is identified in the same region. The incidence of conventional dysplasia, carcinoma in-situ, and invasive carcinoma were recorded and correlated with the cell types and size of the lesion.

### Statistical Analysis

Statistical analysis was performed using IBM Corp. Released 2017. IBM SPSS Statistics for Windows, Version 25.0. Armonk, NY: IBM Corp. The normality of continuous variables was investigated by the Shapiro-Wilk’s test. Descriptive statistics were presented using mean and standard deviation for normally distributed variables and median (and minimum-maximum) for the non-normally distributed variables. Non-parametric statistical methods were used for values with a skewed distribution. The Mann-Whitney U test was used for the comparison of two non-normally distributed groups. The Kruskal-Wallis test was used or the comparison of three non-normally distributed groups. The χ² test (Fisher Exact test where available) was used for categorical variables and expressed as observation counts (and percentages). Statistical signiﬁcance was accepted when the two-sided p value was lower than 0.05.

## RESULTS

### Clinical Findings

The clinical findings of the patients with these tumors are shown in Table 1. The vast majority of the patients were male with a male to female ratio of 70/8. The male predominance decreased with increasing size of the condyloma, with a male/female ratio of 14/1 in the group with size 2-5 cm, and 5.5/1 in the group with tumors measuring 5-10 cm, and dropped down to 2/1 (p=0.08) in cases with tumors larger than 10 cm. The overall mean age was 38 years (range: 20-66). The giant (>10 cm) condylomas occurred in patients nearly a decade older (mean age 46 vs. 38, p=0.09).

**Table 1:** Table I: Distribution of age, gender, dysplasia, invasion and HIV status by condyloma size.

**Tumor Size**	**2-5 cm (n=59) (75%)**	**5-10 cm (n=13) (17%)**	**>10 cm (n=6) (8%)**	***p*** **values**
**Age (mean ±SD)**	38 (±11.8)	37 (±12.3)	46 (±11.3)	0.09
**Gender (Male/Female)**	56/3	11/2	4/2	0.08
**Carcinoma in-situ [n (%)]**	4 (6.7%)	0 (0%)	0 (0%)	
**Invasion [n (%)]**	5 (8.5%)	3 (23%)	3 (50%)	0.02
**Known HIV (+) (%) **	16 (27%)	3 (23%)	1 (17%)	0.4

**HIV:** Human immunodeficiency virus.

### Pathologic Findings

**Histologic subtypes: **Two distinct subsets of condyloma acuminata were recognized by the morphology of the lining epithelium: epidermal and mucosal. 74% of the cases were classified in one of these two categories. The characteristic histomorphologic findings of these categories were as follows:

**1) Epidermal type (50%)** recapitulated the characteristics of the epidermis with a well-defined granular layer, distinct stratum corneum, and decreased glycogen in the cells. Half of the cases (50%) were of this (epidermal) type. If taken in isolation and examined at high-power, this type could be indistinguishable from the normal epidermis. High-grade dysplasia was identified in 13% of cases and invasive carcinoma in 8% from this epidermal subtype.

**2) Mucosal type (50%):** This type of condyloma has an inconspicuous stratum corneum with prominent koilocytic changes. High-grade dysplasia was identified in 2.5% and invasive carcinoma was seen in 20.5% of this condyloma subtype. Although the frequency of invasive carcinoma appeared to be higher in the mucosal type cases, this did not reach statistical significance (p=0.7).

**Figure 1:**
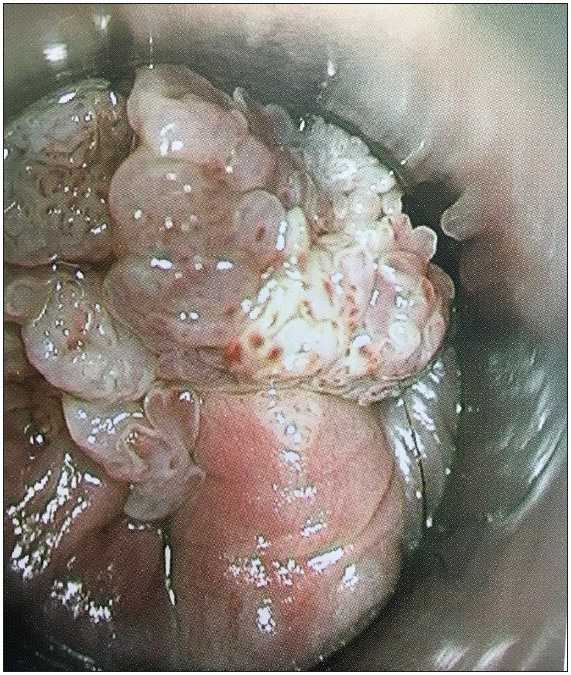
The endoscopic examination of anal condyloma reveals a polypoid, cauliflower-shaped lesion.

**Figure 2:**
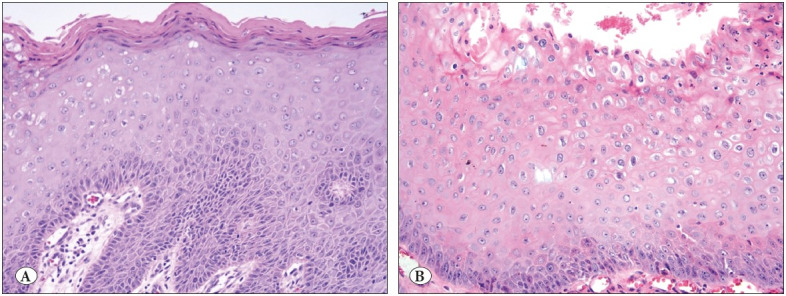
The two distinct subsets of condyloma: **A)** Epidermal, harboring a distinct granular layer, stratum corneum, and decreased glycogen in the cells. If observed in isolation at high-power, this type could be indistinguishable from normal epidermis (H&E; x200). **B)** Mucosal, with an inconspicuous stratum corneum and prominent koilocytic changes (H&E; x200).

Endoscopic appearance and histologic subtypes are seen in Figure 1 and Figure 2

**Patterns of dysplasia: **All cases were reviewed for patterns of dysplasia that were distinct from conventional. Three distinct patterns of dysplasia were identified: basaloid, dyskeratotic, and giant cell-type.

**1) Basaloid:** The basaloid phenotype demonstrated an expansion of basal or parabasal-like cells towards the upper layers of the mucosa with readily identifiable mitoses. This type of dysplasia recapitulated what was seen in a category of anal intraepithelial neoplasia with advanced progression preferably designated as *squamous cell carcinoma of basaloid type *(6). This type of dysplasia was identified in 12% of the cases (n=10), nine of which were in the medium-large group and one in the large group. Eight of the nine cases that had isolated high-grade dysplasia were of the basaloid type. Invasive carcinoma was present in three of the cases with this type of dysplasia.

**2) Giant-cell type of dysplasia** was characterized by bizarre large, highly pleomorphic nuclei, often with multinucleated giant cells, and typically occurring in relatively mature, squamous mucosa as dispersed individual cells. This type of dysplasia was identified in 5% of the cases; two cases were present in the medium-large group, and one case each was present in the large and giant groups. One of the nine cases with isolated high-grade dysplasia was of the giant cell type. Three of the cases with this type of dysplasia had associated invasive carcinoma, two of which also had concurrent dyskeratotic dysplasia (see below, group 3).

**Figure 3:**
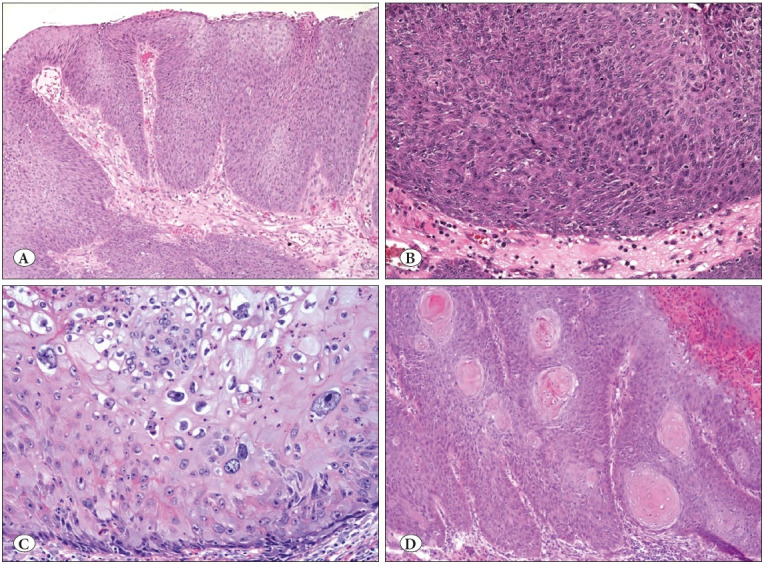
**A-B)** Basaloid type of dysplasia, showing expansion of basal or parabasal-like cells towards the upper layers of the mucosa with readily identifiable mitoses. (H&E; x40&200). **C)** Giant-cell type dysplasia, characterized by large pleomorphic nuclei (H&E; x400). **D)** Dyskeratotic dysplasia, characterized by keratinizing nests (H&E; x40).

**3) The dyskeratotic form of dysplasia** was characterized by groups of or individual cells with inappropriate maturation relative to its level within the epithelium. This dys-maturation often manifested as zones of densely acidophilic cells. Dyskeratotic single cells and overtly keratinizing nests were seen. Dyskeratotic dysplasia was present in 6 cases (8% of all cases): 3 cases from the medium-large group, one from the very large group and two from the giant group. This subtype was commonly seen in association with conventional well-differentiated squamous cell carcinoma (5 cases) and one case of high-grade dysplasia. Patterns of dysplasia are seen in Figure 3.

### Incidence of dysplasia, carcinoma in-situ and invasive squamous cell carcinoma

***Medium-large group (2-5 cm):*** This group included 59 cases (75% of all cases), 56 males and 3 females with a mean age of 38 years (±11.8). Among these cases, high-grade dysplasia was identified in 11 cases (18.6%), carcinoma in-situ in 4 cases (6.7%) and invasive squamous cell carcinoma in 5 cases (8.5%). Sixteen patients (27.1%) were known to be HIV positive.

***Large group (5-10 cm):*** This group included 13 cases (17% of all cases), 11 males and 2 females with a mean age of 37 years (±12.3). Among these cases, high-grade dysplasia and carcinoma in-situ were not identified but invasive squamous cell carcinoma was seen in 3 cases (23%). Three patients (23%) were known to have a positive HIV status.

***Giant group (>10 cm):*** This group included 6 cases (8% of all cases), 4 males and 2 females with a mean age of 46 years (±11.3). Among these cases, high-grade dysplasia and carcinoma in-situ were not identified but invasive squamous cell carcinoma was seen in 3 cases (50%). One of the patients was HIV positive (16.7%).

**Figure 4:**
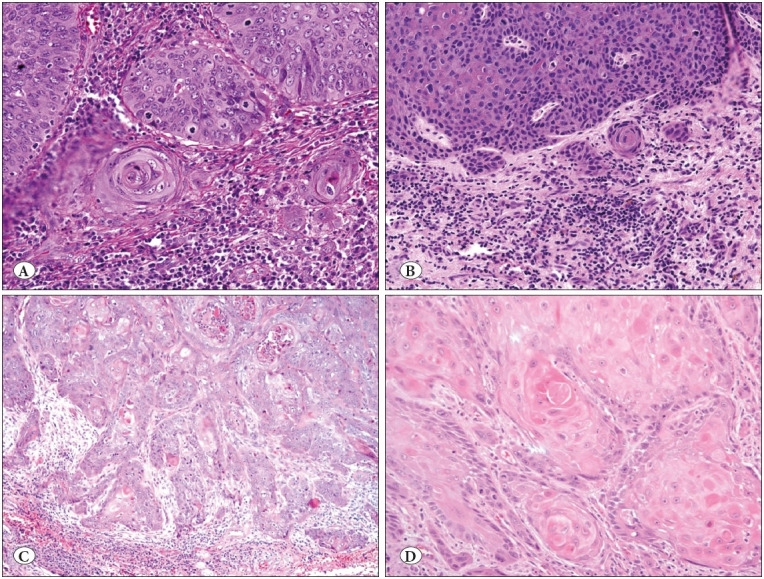
**A)** Condyloma acuminata with focal microinvasion (H&E; x200). **B)** Condyloma acuminata with high-grade dysplasia and focal microinvasion highlighted by a cuff of chronic inflammation (H&E; x200). **C-D)** Condyloma acuminata with invasive squamous cell carcinoma [...] arising in keratinizing type of dysplasia (H&E; x100 & x200).

***Overall incidence:*** The incidence of conventional high-grade dysplasia in the absence of an associated squamous cell carcinoma was 11.2% (n=9), all of which occurred within the 2-5 cm category. Three of these cases were HIV positive. The incidence of invasive carcinoma was 14% (n=11). There was a statistically significant trend (*p*=0.02) for increasing incidence of invasion with size. Seven (63% of all invasive carcinomas) cases from the invasive carcinoma category were microinvasive, penetrating <3 mm below the basement membrane of the condyloma. Four cases of invasive squamous cell carcinoma penetrating greater than 3 mm were distributed as follows: Medium-large: 1 case, Large: 2 cases, and Giant: 1 case. Invasive carcinoma patterns are seen in Figure 4.

## DISCUSSION

Since Buschke and Löwenstein’s description in 1925, it has been well-established that GCAs harbor the potential for malignant transformation (3,7–9). The anal condylomas reported as “giant” in the literature, ranged from 1.5 cm to 30 cm in maximum dimension (7). Consequently, the question arises: Are giant condylomas a morphologic variant of verrucous carcinomas or are they distinct entities? (1). Some believe that they are the same entity, using the terms GCA and verrucous carcinoma interchangeably (4), whereas others speculate that GCAs and verrucous carcinomas represent distinct entities with divergent mechanisms for pathogenesis (10,11). There is some credence to the latter viewpoint because it appears that verrucous carcinomas usually do not arise from transformation of a pre-existing condyloma, although exceptions have been reported (12). Instead, it is a well-differentiated, cytologically bland, low-grade squamous cell carcinoma with broad, pushing borders and local invasion (1). Conversely, GCAs share analogous histologic features with the conventional condyloma mentioned earlier. Kraus and Perez-Mesa proposed that condyloma acuminatum, GCA, verrucous carcinoma, and squamous cell carcinomas lie on the same pathologic continuum (13). Clinically, there are a number of similar treatment options for GCAs and verrucous carcinomas, including local surgical excision, chemotherapy and radiation, depending on the extent of disease (3,14,15).

This study presents the largest series-to-date subjecting large and giant anal condylomas, their clinicopathologic analysis and histological classification. The striking male predominance (overall M/F: 70/8, with a tendency to decrease with increasing size of the lesion) and young age were remarkable features, along with the documented positive HIV status in 25% of the patients. It is possible that some of the remaining cases may also have been HIV positive but the testing and other information was not available to the authors.

Two distinct histological subsets of anal condylomas were discerned in this study: epidermal and mucosal, however, overlaps occurred in nearly 1/4 of the cases. The main significance of this classification is the need of recognizing different morphological aspects of these lesions, especially in small and fragmented biopsies. Epidermal type, a form of condyloma/dysplasia not recognized previously, is virtually indistinguishable from normal epidermis. The design of the epithelium, the texture of the cytoplasms, the relationship of the cells with each other, and the layers were characteristic of skin. In fact, in many areas, a granular layer was also noted, completing the picture of epidermis. Because of its close resemblance to normal epidermis, these areas would have been almost impossible to recognize as abnormal, let alone as a part of condyloma, if they were taken in isolation on high-power examination. However, they were lining florid condylomatous lesions, proving their pathologic nature. We have noted this type of lesion in the mucosa of oral cavity and it has been illustrated in some publications. Recently, a group of cases were reported in the esophagus under the heading of “esophageal epidermoid metaplasia”, and are believed to be early precursor lesions (16). Our study proves that there is indeed epidermal-like neoplastic transformation of mucosal sites. Therefore, this epidermal-type of condyloma elucidated in this study is not only significant diagnostically as a subtle form of dysplasia, but also important in terms of proving that epidermal-like dysplasia exists as a concept. Moreover, there were differences in the clinicopathologic associations of the mucosal versus epidermal types of dysplasia. The incidence of high-grade dysplasia was higher in epidermal-type condyloma (13% vs. 2.5%), whereas the incidence of invasive squamous cell carcinoma was higher in mucosal-type condyloma (20.5% vs. 8%, respectively).

In this study, three distinct types of dysplasia were also observed in large anal condylomas: basaloid, giant cell and dyskeratotic types. Among those, dyskeratotic dysplasia was seen in association with squamous cell carcinoma and its presence in a biopsy should raise suspicion for an invasive lesion. In fact, it may be better to regard it as “surface” component of a keratinizing squamous cell carcinoma and evaluate the case accordingly.

The overall incidence of invasive carcinoma in anal condylomas in this study was 14% There was a statistically significant trend (p=0.02) for increasing incidence of invasion with size; however, there was no discernable trend in depth of invasion relative to condyloma size. More importantly, although half of the giant condylomas displayed invasion, the majority was microscopic. This may be highly pertinent to the biology of these lesions. In essence, Löwenstein-Bushcke disease can be regarded as a virally driven adenomatous (“papilloma”-type) tumor of the anal squamous mucosa. As such, these can be regarded as tumoral intraepithelial neoplasia. Similar to most tumoral intraepithelial neoplasms, the neoplastic cells in this disease somehow tend to grow in the surface, eventually forming exophytic tumors without invading the stroma. In other words, akin to the dichotomy that is well known in urothelial neoplasms (17), there appears to be a dichotomy in the HPV carcinogenesis in the anus as well. In the urothelium, it has been demonstrated amply that the papillary urothelial neoplasms represent a different pathway of carcinogenesis than the “flat” carcinoma in-situ pathway, in terms of molecular background, progression rate, and clinical characteristics (18). The former has a much more indolent, protracted clinical course.

Some authors regard Löwenstein-Bushcke as a verrucous-type squamous carcinoma (19). Verrucous carcinoma is a tumor that is well-recognized in the upper aerodigestive tract. Similar to the tumoral intraepithelial neoplasms, it continues to grow exophytically, before showing conventional invasive carcinoma (20). However, verrucous carcinoma appears to be something in between these two, a special type of invasive carcinoma rather than being a purely pre-invasive neoplasm. Characteristically, it has bulbous edges that represent broad-based pushing-type infiltration, and that is why it can show destructive behavior, even without showing regular type invasiveness. In fact, it may be better to regard verrucous carcinoma as a form of invasive carcinoma that has “blunt” invasion. In the case of Löwenstein-Bushcke, the process has all the characteristics of an ordinary condyloma acuminata, but a very large one. In most cases, one does not get the impression of a pushing-border invasion that is characteristic of verrucous carcinoma. In fact, in the case of epidermal-type, they do not even show much cytologic atypia. Of note, the fact that most of the dysplastic changes occurred in medium-size cases may signify a different branching that takes place during the advancement of these large condylomas, with some cases acquiring conventional dysplastic changes. On the other hand, the frequency of invasive carcinoma increases by size. However, this is kind of expected, because after all, the probability of changes that lead to invasion increases with the number of neoplastic cells that are present. In this study, no funds were available to conduct specific HPV typing. It would be an interesting next step to investigate the associations of different HPV types with the histopathologic observations elucidated in this study. Additionally, since clinical follow up data was limited, no inferences could be made regarding the direct prognostic associations of some of the histomorphologic observations.

In summary, it is advisable to further categorize anal condylomas (≥2 cm) based on their size, due to the increasing frequency of invasion in larger tumors. Condylomas that are >10 cm seemed to occur in older patients, suggesting a slower growing process. Our findings suggest that Löwenstein-Buschke lesions are mega versions of conventional condyloma. Being verrucoid, large and minimally invasive, they can be regarded as a form of verrucous carcinoma, but they do not display the histologic characteristics of verrucous carcinoma as defined in the aerodigestive tract. They exhibit two types of linings, the mucosal-type that often shows koilocytic changes, and the epidermal-type that can be difficult to recognize in biopsies. Different types of high-grade dysplastic changes that occur in these lesions (basaloid, keratinizing and giant cell) also appear to have distinct behavioral characteristics. These lesions may be associated with invasive carcinoma, albeit limited in amount.

## CONFLICT of INTEREST

The authors have no conflicts of interest or financial ties to disclose.

## FUNDING

No funding was used.

## AUTHORSHIP CONTRIBUTIONS

Concept: **OT,** Design: **OT, MR, OB,** Data collection or processing: **BP, TF, ML,** **TT,** **SB, **Analysis or Interpretation: **BP, MR, TF,** **ML, TT, SB, JA, AA, OB, **Literature search: **AA, **Writing: **OT, TF,** **VA , **Approval: **OB, VA.**
